# A brief review of the latest pharmacological treatments of COVID-19

**DOI:** 10.22088/cjim.11.0.460

**Published:** 2020

**Authors:** Seyed Parsa Eftekhar, Sohrab Kazemi, Ali Akbar Moghadamnia

**Affiliations:** 1 Student Research Committee, Babol University of Medical Sciences, Babol, Iran; 2 Cellular and Molecular Biology Research Center, Health Research Institute, Babol University of Medical Sciences, Babol Iran

**Keywords:** COVID-19, SARS-CoV-2, 2019-nCoV, pharmacological, treatment, vaccine

## Abstract

New Coronavirus which is called 2019-nCoV (2019-Novel-Coronavirus) or SARS-Cov-2 (Severe Acute respiratory Syndrome-Coronavirus 2) causes deadly pneumonia that first appeared in December 2019 in Wuhan city in China. This virus spreads all over the world quickly and made several problems for the community and healthcare system. Several drugs have been tried to manage COVID-19; however, our knowledge of this virus is not complete. At any rate, effective treatment or vaccine for this disease has not been discovered yet. Furthermore, to achieve this goal, more studies are needed on the structure of the virus and its pathogenesis mechanism. In this article, we summarized several articles suggesting treatments of COVID-19.

## Introduction

Coronaviruses cause infection of the respiratory tract. Moreover, some strains of Coronaviruses like SARS (Severe Acute Respiratory Syndrome) and MERS (Middle East Respiratory Syndrome) have high mortality and infectivity that bring about damage to public health and industries as we saw in history (1). New Coronavirus which is called 2019-nCoV (2019-Novel-Coronavirus) or SARS-Cov-2 (Severe acute respiratory Syndrome-Coronavirus 2) causes deadly pneumonia that first appeared in December 2019 in Wuhan City in China (2). This virus is now spreading quickly in the world as well as the person-to-person transmission which is studied in some papers (3). COVID-19 may be an asymptomatic disease, but in most cases, its clinical manifestations vary from mild to severe, and even cause hospitalization and mortality. The most common clinical symptoms are fever and cough. Other symptoms like myalgia and dyspnea are observed not as common as fever and cough (4, 5). The prevalence of clinical symptoms in adults are more obvious than children (6). Mainly laboratory findings include hypoalbuminemia, and increased inflammatory markers levels like C-reactive protein, LDH and ESR. Based on meta-analysis conducted by Rodriguez-Morales, A. J., et al lymphopenia was found in more than 40% of patients (6). According to the research implemented by C. Huang, .increased cytokines levels such as IL1B, IFNγ, IP10, MCP and other cytokines probably activate T-helper-1 (Th1) cell. Furthermore, T-helper-2 (Th2) cells secrete IL4 and IL10. It seems that the pathways have roles in COVID-19 pathophysiology, but as far as we know, further studies are necessary in this field (5). Given that vaccine production for COVID-19 is time-consuming, it has also deleterious effects on all aspects of society. It should be focused on treating the disease simultaneously with vaccine production. The vaccine can help prevent the spread of the disease, but separate treatment is needed to treat people infected with the virus. In this article, we summarized several articles suggesting treatments of COVID-19. 

**Fusion and entry inhibitors**

**1. Hydroxychloroquine sulphate and chloroquine phosphate: **Chloroquine phosphate, a widely-used antimalarial and autoimmune disease drug is used as a treatment in viral diseases 20 years ago since its anti-viral effects were observed in vitro (7). A study was conducted on 42 hospitalized SARS-CoV-2 infected patients with inclusion criteria: age>12 and positive nasopharyngeal samples PCR-test and patients were followed-up for 14 days (8). 

Thirty-six patients received oral hydroxychloroquine sulphate, whereas 16 patients were the control group. The mean age of hydroxychloroquine-treated patients was more than the control group (51.7 years’ *vs* 37.3 years). Hydroxychloroquine-treated patients received 200 mg hydroxychloroquine orally, three times a day. Additionally, azithromycin was added to the medication of 6 hydroxychloroquine-treated patients (500 mg on day 1 and should be continued with 250 mg for four days). On day 6, the proportion of positive PCR-test between hydroxychloroquine-treated and control group was significantly different (70% in hydroxychloroquine-treated vs 12.5% in the control group). Furthermore, all patients who received azithromycin and hydroxychloroquine sulphate had a negative PCR-test compared to patients treated with hydroxychloroquine sulphate alone (57.1%) (8).

According to Yao, X., et al.’s study, although hydroxychloroquine has a more potent effect in comparison to chloroquine, both have good antiviral effects in vitro and inhibit SARS-CoV-2 replication. (hydroxychloroquine EC_50_ < chloroquine EC_50_) (9). Yao, X., et al suggested the treatment should be initiated by oral hydroxychloroquine sulfate 400 mg two times on the first day and pursued by 200 mg two times in a day for 4 more days (9). In addition to the above mechanism, hydroxychloroquine sulfate has an immunomodulatory effect. Based on the observation by Huang, C., et al., IL-6 and IL-10 raise in response to SARS-CoV-2 and cytokine storm may play a role in the pathophysiology of the disease. It seems hydroxychloroquine and chloroquine can suppress these pathways because of their immunomodulatory effect (5). We will discuss in more detail about cytokine secretion inhibitors in the following paragraphs. 

**2. ACE2 related therapies: **Novel coronavirus enters cells using SARS-CoV receptor ACE2 (10, 11). Moreover, novel coronavirus uses transmembrane protease serine 2 (TMPRSS2) for priming spike (S) protein. Based on the above evidence, camostat mesylate that is a serine protease inhibitor can be used as a treatment for COVID-19 (10). According to the role of ACE2 in the pathophysiology of disease and experiences about SARS-CoV (12) suggested blocking ACE2 receptor or delivering excessive soluble form of ACE2 can be used as a treatment against novel coronavirus (12).

**3. Endocytosis inhibition: **Endocytosis might be a mechanism which novel coronavirus enters cells. AKK1 (AP2-associated protein kinase 1) plays a role in endocytosis regulation. AKK1 inhibitors like barcitinib may be effective against novel coronavirus. Richardson, P., et al. suggested oral barcitinib on therapeutic dosing (2 mg or 4 mg once daily) could be examined in COVID-19 patients as drug (13).

**4. Other drugs: **L. Deng et al. evaluated arbidol or umifenovir as a treatment for 2019-nCoV. In this study, 16 patients were treated with oral arbidol and lopinavir/ritonavir against 17 patients treated with lopinavir/ritonavir monotherapy. The number of patients with negative nasopharyngeal specimen was more in combination therapy after 7 and 14 days. Moreover, the improvement of chest CT scan in combination therapy was faster than the monotherapy group. The above evidence points to the efficacy of arbidol as an effective treatment for COVID-19. Also, 43% of patients in the monotherapy group received corticosteroid, whereas 31% of them had improvement in chest CT scans, which may indicate that corticosteroids did not affect the disease improvement (14).

Teicoplanin is an antibiotic used as a treatment for staphylococcal infections. Moreover, teicoplanin showed an antiviral effect against MERS. J. Zhang et al., reported teicoplanin can inhibit SARS-CoV-2 in vitro and must be considered as a treatment for COVID-19. However, further investigations are needed in this field (15, 16) Table 1. 

**Cytokine secretion suppressors**

**Corticosteroids: **Huang, C., et al. reported high levels of cytokines in COVID-19 can be suppressed by corticosteroids, but this treatment can be used for severe illness and it should not be used routinely for all patients (5).

Wang, Y., et al evaluated corticosteroid effects on clinical, radiographic and laboratory parameters in 46 patients with COVID-19 who had severe pneumonia. Intravenous methylprednisolone (1 to 2 mg/kg/day) was administered for 26 patients. Moreover, antiviral therapy (lopinavir/ritonavir), supplemental oxygen therapy, antibacterial therapy and immunoenhancement therapy were administered for all of the patients. As a result, the duration of returning body temperature to the normal state was shorter in the corticosteroid-treated group. Moreover, the absorption degree of focus in the lung in the CT scan improved faster in the corticosteroid-treated group. Also, SpO_2_ increased faster in the corticosteroid-treated group. However, laboratory parameters had no significant differences between the two groups (17). Zhou, W., et al. assessed corticosteroid therapy on 15 patients with COVID-19 in critical status admitted to ICU. They suggested low dose and short durational corticosteroid therapy (methylprednisolone, <1 mg/kg/day, less than seven days) with monitoring of adverse effects could be beneficial for severe novel coronavirus pneumonia. However, this treatment does not affect survival or mortality, but it can enhance oxygen saturation (SaO_2_) (18). On the other hand, Russell, C. D., et al. suggested corticosteroid therapy should not be recommended because there is no strong evidence confirming corticosteroid therapy is helpful for COVID-19 patients. Moreover, it could have adverse effects on disease progression based on experience from influenza, SARS and MERS (19).

**Table 1:** Antivirals included in the Guidelines for treatment of COVID-19

Treatment	Result	Reference
**Hydroxychloroquine sulphate (200 mg three times a day) + Azithromycin (500 mg on day 1 and 250 mg for the next four days)**	Negative PCR-test in all patients after 6 days	Gautret, P., et al. 2020
**Hydroxychloroquine sulphate (suggested dose: 400 mg two times on the first day and pursued by 200 mg two times in a day for 4 more days)**	Good antiviral effects in vitro	Yao, X., et al. 2020
**Camostat mesylate**	As a serine protease inhibitor could be protective against virus entry to cell (need further studies)	Hoffmann, M., et al. 2020
**Barcitinib ** **(suggested dose: 2 mg or 4 mg once daily, should be examined)**	As an endocytosis inhibitor could be protective against virus entry to cell	Richardson, P., et al. 2020
**Umifenovir (arbidol)**	More negative nasopharyngeal specimen and faster chest CT-scan improvement in umifenovir + lopinavir/ritonavir group against lopinavir/ritonavir monotherapy	Deng, L., et al. 2020
**Teicoplanin**	Good antiviral effects in vitro (need further studies)	Zhang, J., et al. 2020
**Methylprednisolone** **(Intravenous: 1 to 2 mg/kg/day)**	Faster improvement of chest CT-scan parameters and body temperature in severe pneumonia cases	Wang, Y., et al. 2020
**Methylprednisolone** **(<1 mg/kg/day, ** **less than seven days)**	Enhance oxygen saturation in severe pneumonia cases but this therapy does not affect mortality or survival	Zhou, W., et al. 2020
**Ribavirin, Penciclovir and Favipiravir**	High concentration of these three nucleoside analogs are needed to reduce SARS-CoV-2 infection in vitro	Wang, M., et al. 2020
**Nitazoxanide**	It can inhibit SARS-CoV-2 infection in low-micromolar concentration in vitro	Wang, M., et al. 2020
**Remdesivir, Chloroquine phosphate**	These drugs significantly inhibit SARS-CoV-2 infection in low-micromolar concentration in vitro	Wang, M., et al. 2020
**Ribavirin, Remdesivir, Sofosbuvir, Galidesivir and Tenofovir**	Strong binding to RdRp model of SARS-CoV-2 and may be effective (need further studies)	Elfiky, A. A. 2020

**Replication inhibitors: **Based on protocol by Indian Council of Medical Research, lopinavir/ritonavir 200 mg/50 mg tablets given two times a day for 14 days or for 7 days after becoming asymptomatic is recommended. Moreover, 400 mg lopinavir/100 mg ritonavir (5 ml suspension, atomization inhalation) twice daily for 14 days or for 7 days after becoming asymptomatic for patients who cannot take medication orally is suggested (20). Wang, M., et al. evaluated five FDA-approved drugs included ribavirin, penciclovir, nitazoxanide, nafamostat and chloroquine along with two antiviral drugs counted, remdesivir and favipiravir against a clinical isolate SARS-CoV-2 in (20).

Based on Wang, M., et al.’s study, a high concentration of three nucleoside analogs included ribavirin, penciclovir and favipiravir are needed to reduce SARS-CoV-2 infection as well as nitazoxanide that is an antiprotozoal compound can inhibit SARS-CoV-2 infection in low-micromolar concentration (20). Additionally, Wang, M., et al. showed remdesivir and chloroquine phosphate significantly inhibited SARS-CoV-2 infection in vitro in low-micromolar concentration (20). According to Elfiky, A. A.’s study, 5 FDA approved anti-polymerase drugs that include ribavirin, remdesivir, sofosbuvir, galidesivir and tenofovir have a strong binding characteristic to RNA-dependent RNA polymerase (RdRp) model of the novel coronavirus. So, these drugs can be used as effective drugs against novel coronavirus (21).

**Vaccine: **Vaccine is a proper and effective tool for preventing and controlling viral infections. Various methods are currently being studied and evaluated for COVID-19 vaccine production. Moreover, produced vaccines need several clinical trials on animals and humans. Vaccine safety is an important issue, and vaccines should not be produced without regard to safety (22). Also, vaccine production requires knowledge about various aspects of the virus including virus structure, mechanism of pathogenicity and body’s immune response to the virus. There are several difficulties in vaccine production; for instance, virus mutagenicity is one of them. Currently, two subtypes including L and S are known. In addition, these subtypes also differ in the transmission ability and the severity of the pathogenesis (23). As mentioned, more studies are needed on vaccine production. 

**Treatment Protocols for Novel Coronavirus Pneumonia: **National Health Commission & State Administration of Traditional Chinese Medicine on March 3, 2020, suggested "antiviral treatment including α-interferon (5 million U or equivalent dose each time for adults, adding 2ml of sterilized water, atomization inhalation twice daily), lopinavir/ritonavir (200 mg/50mg per pill for adults, two pills each time, twice daily, no longer than 10 days), ribavirin (suggested to be used jointly with interferon or lopinavir/ritonavir, 500 mg each time for adults, twice or three times of intravenous injection daily, no longer than 10 days), oral chloroquine phosphate (500 mg bid for 7 days for adults aged 18-65 with body weight over 50 kg; 500 mg bid for days 1&2 and 500 mg qd for days 3-7 for adults with body weight below 50 kg), oral arbidol (200 mg tid for adults, no longer than 10 days)" (24).

Moreover, the improper use of antibiotics, especially broad-spectrum antibiotics, should be avoided. Also, immunotherapy with tocilizumab could be useful in patients with extensive lung injury and critical patients with increased levels of IL-6. Corticosteroid therapy should be used just for a short duration in patients with progressive worsening of oxygen levels along with extreme stimulation of the body’s inflammatory response (24).

NIH recommends remdesivir as antiviral treatment in severe cases (patients with SpO2≤94%, or those need supplemental oxygen, or mechanical ventilation, or extracorporeal oxygenation) but it is not recommended for mild or moderate cases. There is no strong evidence about using or not using of chloroquine or hydroxychloroquine, but high-dose chloroquine (600 mg twice daily for 10 days) is not recommended. Furthermore, a combination of hydroxychloroquine and azithromycin (because toxicity) is not recommended. NIH does not suggest lopinavir/ritonavir or other HIV protease inhibitors (because of undesirable pharmacodynamics) (25). Besides, there is no strong evidence about the application or not application of COVID-19 convalescent plasma or SARS-CoV-2 immune globulins. Also, non-SARS-CoV-2 specific intravenous immune globulin is not recommended to use, except in a clinical trial. There is no strong evidence about prescribing or not prescribing of interleukin-1 inhibitors like anakinra and interleukin-6 inhibitors like sarilumab, siltuximab, and tocilizumab. Furthermore, interferons and janus kinase inhibitors like barcitinib are not recommended (25). 

In this survey, we briefly reviewed several studies on COVID-19 treatments. Studies in humans and in vitro have shown the efficacy of many drugs on novel coronavirus; however, no significant vaccine and treatment for 2019-nCoV has been reported. Some researchers reported hydroxychloroquine sulphate as an effective drug against COVID-19 but nowadays, it is not clear that hydroxychloroquine and chloroquine are practical or not. On the other hand, there is controversy about the use of corticosteroids. However, majority agree with the use of corticosteroids in critically ill patients. As we mentioned above, based on the mechanism of entry of the virus into the cell, several drugs have been suggested. Due to the lack of vaccines along with the high rate of the outbreak of this disease, treatments for this disease are needed as soon as possible. Finally, comprehensive studies are needed to ensure the effectiveness of the above-mentioned drugs with more case studies and in vitro experiments.

## Conflict of Interests:

There are no Conflict of Interests.
